# Co-Ultramicronized Palmitoylethanolamide/Luteolin Promotes Neuronal Regeneration after Spinal Cord Injury

**DOI:** 10.3389/fphar.2016.00047

**Published:** 2016-03-08

**Authors:** Rosalia Crupi, Daniela Impellizzeri, Giuseppe Bruschetta, Marika Cordaro, Irene Paterniti, Rosalba Siracusa, Salvatore Cuzzocrea, Emanuela Esposito

**Affiliations:** ^1^Department of Biological and Environmental Sciences, University of MessinaMessina, Italy; ^2^Manchester Biomedical Research Centre, Manchester Royal Infirmary, School of Medicine, The University of ManchesterManchester, UK

**Keywords:** luteolin, neurogenesis, palmitoylethanolamide, regeneration, spinal cord injury

## Abstract

Spinal cord injury (SCI) stimulates activation of astrocytes and infiltration of immune cells at the lesion site; however, the mechanism that promotes the birth of new neurons is still under debate. Neuronal regeneration is restricted after spinal cord injury, but can be stimulated by experimental intervention. Previously we demonstrated that treatment co-ultramicronized palmitoylethanolamide and luteolin, namely co-ultraPEALut, reduced inflammation. The present study was designed to explore the neuroregenerative properties of co-ultraPEALut in an estabished murine model of SCI. A vascular clip was applied to the spinal cord dura at T5–T8 to provoke injury. Mice were treated with co-ultraPEALut (1 mg/kg, intraperitoneally) daily for 72 h after SCI. Co-ultraPEALut increased the numbers of both bromodeoxyuridine-positive nuclei and doublecortin-immunoreactive cells in the spinal cord of injured mice. To correlate neuronal development with synaptic plasticity a Golgi method was employed to analyze dendritic spine density. Co-ultraPEALut administration stimulated expression of the neurotrophic factors brain-derived neurotrophic factor, glial cell-derived neurotrophic factor, nerve growth factor, and neurotrophin-3. These findings show a prominent effect of co-ultraPEALut administration in the management of survival and differentiation of new neurons and spine maturation, and may represent a therapeutic treatment for spinal cord and other traumatic diseases.

## Introduction

Spinal cord injury (SCI) results in long-term disability, and presents enormous social and health costs to the patient and family (Chiu et al., 2010). The annual incidence of SCI is 15–40 cases per million and is particularly prevalent in young males, the most frequent cause being vehicular accidents (Iijima et al., 2009). SCI is frequently associated with permanent modifications in strength, sensation, and other body functions below the site of injury (Coleman and Geisler, 2004). Furthermore, SCI induces tissue damage characterized by local cellular and biochemical reactions that in turn contribute to neurological dysfunction (Yuan et al., 2013). At the same time, SCI is characterized by a natural neuroplasticity which can occur some weeks or months after the original insult, stimulating some functional recovery (Darian-Smith, 2009; Kernie and Parent, 2010). This endogenous repair mechanism remains unclear, but may comprise dendritic remodeling, axonal sprouting, as well as local neuronal circuit reorganization and remyelination (Darian-Smith, 2009). In this context, one possible regenerative mechanism may be neurogenesis (Kazanis, 2009). Neural stem and progenitor cells stimulate a dynamic process known as adult neurogenesis. These cells are located in two specific “neurogenic” brain regions, the subventricular zone (SVZ) along the margin of the lateral ventricle (Alvarez-Buylla and Garcia-Verdugo, 2002) and the subgranular zone (SGZ) of the hippocampal dentate gyrus (Seaberg and Van Der Kooy, 2002). Several physiological, pathological, and pharmacological stimuli are able to modulate adult neurogenesis. In other adult central nervous system (CNS) regions, neurogenesis is believed to be limited under physiological conditions, but could be induced after injury (Gould, 2007).

In addition to these two brain regions, other niches rich in markers of morpho-functional neuroplasticity contain adult neural stem cells, but with lower levels compared to the SVZ and SGZ. One of these, namely, the hindbrain dorsal vagal complex (Bauer et al., 2005; Moyse et al., 2006) is responsive to injury since vagotomy induces both microgliosis and astrogliosis in this area (Bauer et al., 2005). In physiological conditions the adult mammalian spinal cord is believed to lack neurogenic areas. Despite containing multipotent neural stem cells that *in vitro* could generate functional neurons (Meletis et al., 2008), their potential is mainly restricted to the glial lineage. Following SCI, astrocytes become reactive and immune cells infiltrate the area adjacent to the lesion (Uchida et al., 2012). Neurogenesis after SCI is related to the localization and severity of injury (Nishimura and Isa, 2009). In this context another important question concerns the distal consequences of SCI in the forebrain. A topographic and neuronal reorganization reportedly occurs after SCI, implying that neuronal responses can take place in the brain in response to a distal SCI (Freund et al., 2011). Indeed, cervical dorsal rhizotomy, which provokes cortical reorganization analogous to that seen after SCI, is able to stimulate neurogenesis in the primary sensorimotor cortex of adult monkeys (Vessal and Darian-Smith, 2010).

Traumatic injury of the spinal cord is also characterized by a neuroinflammatory pathway that is associated with decreased of functional recovery because of the development of cicatrix tissue, as well as necrosis or apoptosis of neurons and oligodendrocytes for at least 2 weeks post-injury; cell loss occurs quickly at the injury site (Cuzzocrea et al., 2001). The complexity of SCI has hampered the identification of pharmacological agents able to ameliorate outcome after spinal cord lesion. Even small anatomical gains can generate disproportionate functional benefits (Blight, 1983), which encourages the possibility that early pharmacological treatments which reduce SCI-associated damage could facilitate clinical outcome. Unfortunately, the current management of SCI is limited to treatment with corticosteroids, supportive care, and spine stabilization.

Our earlier studies focused on a murine spinal cord compression model and the neuroprotective and neuroregenerative properties of a formulation composed of co-ultramicronized palmitoylethanolamide (PEA), an endogenous fatty acid amide signaling molecule together with the flavonoid luteolin (Lut) (co-ultraPEALut; Paterniti et al., 2013b). PEA inhibits peripheral inflammation and degranulation of mast cells (Berdyshev et al., 1998) and displays antinociceptive effects in rodents (Lambert et al., 2002). We previously reported that administration of PEA (10 mg/kg) diminished inflammatory processes in a mouse model of SCI (Genovese et al., 2008) and traumatic brain injury in mice (Ahmad et al., 2012). PEA lacks natural antioxidant activity to arrest the formation of free radicals responsible for the damage to DNA, lipids, and proteins occurring after SCI. Lut, a common flavonoid found in fruits, vegetables, and medicinal herbs has many pharmacological activities (Xu et al., 2010), in particular radical scavenging and cytoprotective properties (Lin et al., 2007). Taking the above as a starting point, we investigated the neuroregenerative effect of co-ultraPEALut in modulating neurogenesis and neuroplasticity in an experimental model of SCI.

## Materials and methods

### Animals

Male CD1 mice weighing 25–30 g (Harlan, Milan, Italy) were housed in a controlled environment, and provided with standard rodent chow and water. Animal care was in compliance with Italian regulations on protection of animals used for experimental and other scientific purposes (DM 116192), as well as with the European Economic Community regulations (OJ of EC L 358/1 12/18/1986). All experimental studies on animals followed internationally recognized guidelines.

### Spinal cord injury

Mice were anesthetized with intraperitoneal (i.p.) administration of ketamine and xylazine (2.6 and 0.16 mg/kg body weight, respectively). A longitudinal incision was made along the midline of the back, exposing the paravertebral muscles, as previously described (Paterniti et al., 2011). These muscles were dissected away, the spinal cord was exposed via a four-level T5–T8 laminectomy and SCI was produced by extradural compression at T6–T7 level, using an aneurysm clip with a closing force of 24 g. In all injured groups, the spinal cord was compressed for 1 min. Sham animals were only subjected to laminectomy. Following surgery, 1.0 cm^3^ of saline was administered subcutaneously in order to replace the blood volume lost during surgery. During recovery from anesthesia, mice were placed on a warm heating pad and covered with a warm towel. The mice were individually housed in a temperature-controlled room at 27°C. Food and water were provided to the mice *ad libitum*. During this time period, the animals' bladders were manually voided twice a day until the mice were able to regain normal bladder function.

### Experimental groups and treatments

Mice were randomly allocated to the following groups:

1. Sham + vehicle (carboxymethylcellulose): mice were subjected to laminectomy but the aneurysm clip was not applied and treated i.p. with vehicle (*n* = 45; *n* = 15 for each experiment).
2. SCI + vehicle: mice were subjected to laminectomy and the aneurysm clip was applied (*n* = 45; *n* = 15 for each experiment).
3. Sham + co-ultraPEALut: mice were subjected to laminectomy but the aneurysm clip was not applied and treated with co-ultraPEALut at a dose of 1 mg/kg i.p. (*n* = 45; *n* = 15 for each experiment).
4. SCI + co-ultraPEALut: mice were subjected to SCI and administered co-ultraPEALut at a dose of 1 mg/kg i.p. daily for 72 h after SCI (*n* = 45; *n* = 15 for each experiment).

Animals were sacrified at 72 h after SCI (Ceruti et al., 2009).

In a separate set of experiments, another 10 animals for each group were observed until 21 days after SCI to evaluate motor score. The co-ultraPEALut group received 1 mg/kg i.p. 30 min after SCI and daily until day 21.

### Behavioral testing

Performance was evaluated before and after SCI with the horizontal grid walking test (Goldshmit et al., 2011). After 2 min of free walking, missteps (normalized to total number of steps taken by the left hind limb) were quantified. Motor function was evaluated 21 days after SCI by the open-field test using the Basso Mouse Scale (BMS) score, as described by Basso et al. (2006).

### Bromodeoxyuridine (BrdU) treatment

In both sham-operated and SCI mice cell proliferation was evaluated by multiple i.p. injections of the thymidine analog BrdU (70 mg/kg), 1 h before SCI, and twice a day (morning and afternoon) up to 72 h after SCI. BrdU incorporation into cell nuclei was assessed by immunohistochemistry.

### Immunohistochemistry

Excised spinal cord were fixed in 10% formaldehyde (w/v)e in phosphate buffered saline (PBS), embedded in Paraplast (Sigma-Aldrich, Milan, Italy). After deparaffinization, sections were incubated for 45 min in PBS containing 10% normal goat serum (Sigma-Aldrich) and 0.1% Triton X-100 (Sigma-Aldrich). Thereafter, 7-μm longitudinal sections were deparaffinized with xylene and rehydrated. BrdU, doublecortin (DCX) and glial fibrillary acidic protein (GFAP) analysis was carried out after boiling in 0.01 M citrate buffer for 4 min. Endogenous peroxidase was quenched with 0.3% (v/v) hydrogen peroxide in 60% (v/v) methanol for 30 min. Non-specific adsorption was minimized by incubating the section in 2% (v/v) normal goat serum in PBS for 20 min. Endogenous biotin or avidin binding sites were blocked by sequential incubation for 15 min with biotin and avidin (DBA, Milan, Italy), respectively. Sections were incubated overnight with: mouse monoclonal anti-BrdU antibody (1:100 in PBS; Santa Cruz, California, USA); goat polyclonal anti-DCX antibody (Santa Cruz, California, USA); mouse anti-MAP2 (microtubule-associated protein 2; 1:1000, Promega, Milan, Italy); mouse anti-GFAP (1:500, Cell Signaling, Danvers, MA, USA). Sections were washed with PBS and incubated with secondary antibody. Specific labeling was detected with a biotinconjugated goat anti-rabbit IgG and avidin-biotin peroxidase complex. Sections were counterstained with nuclear fast red (red background). All sections were observed using light microscopy at 20X magnification (Axostar Plus equipped with Axio-Cam MRc, Zeiss) and analyzed via an imaging computer program (Axio-Vision, Zeiss).

**Scheme 1:**
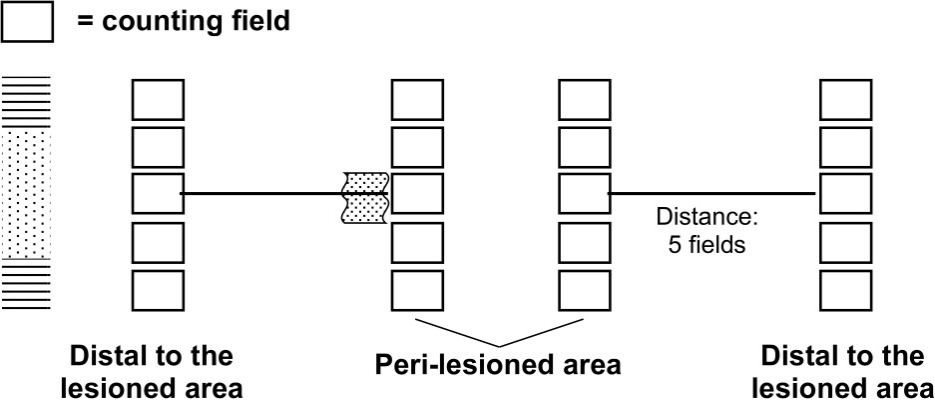
**Design and evaluation of the injured area**.

To quantify BrdU-positive nuclei and DCX-positive cells, for each tissue section at least 10 optical fields in the area at the boundary between the necrotic core and the penumbra (peri-lesioned area) and 10 optical fields distal to the damaged area were counted (for a total of about 1.5 mm^2^, with the five field distance corresponding to about 1.4 mm from the lesion border) as indicated in the Scheme 1 below. Replicates for each experimental condition and histochemical staining were from three different animals. In sham-operated mice, the central area of the corresponding tissue sections were taken as reference point and a comparable number of optical fields counted. Data are expressed as a percentage of total tissue area, as described previously (Shea, 1994).

### Immunofluorescence staining

After deparaffinization and rehydration, detection of NeuN and GFAP was carried out after boiling in 0.1 M citrate buffer for 1 min. Non-specific adsorption was minimized by incubating the section in 2% (v/v) normal goat serum in PBS for 20 min. Sections were incubated with one of the following primary antibodies: mouse monoclonal anti-BrdU (1:100, Millipore), polyclonal rabbit anti-NeuN (1:100, Santa Cruz Biotechnology), rabbit anti-GFAP (1:100, Santa Cruz Biotechnology), or rabbit anti-DCX (1:100, Santa Cruz Biotechnology) in a humidified chamber at 37°C overnight. Sections were washed with PBS and then incubated with either FITC-conjugated anti-mouse Alexa Fluor-488 antibody (1:2000, Molecular Probes, UK) or Texas Red-conjugated anti-rabbit Alexa Fluor-594 antibody (1:1000, Molecular Probes) for 1 h at 37°C. Sections were next washed and nuclei stained with 4′,6′-diamidino-2-phenylindole (2 μg/ml in PBS; Hoechst, Frankfurt; Germany). The perilesional area of each section was observed and photographed using a Leica DM2000 microscope at 20X magnification.

All images were digitalized at a resolution of eight bits into an array of 2560 × 1920 pixels. Optical sections of fluorescence specimens were obtained using a HeNe laser (543 nm), a laser UV (361–365 nm) and an argon laser (458 nm) at a 1-min, 2-s scanning speed with up to eight averages; 1.5-μm sections were obtained using a pinhole of 250. Contrast and brightness were established by examining the most brightly labeled pixels and applying settings that allowed clear visualization of structural details while keeping the highest pixel intensities close to 200. The same settings were used for all images obtained from the other samples that had been processed in parallel. Digital images were cropped and figure montages prepared using Adobe Photoshop CS5 (Adobe Systems; Palo Alto, CA).

### Golgi impregnation

Golgi impregnation was performed according to the directions supplied by FD NeuroTechnologies, FD (FD NeuroTechnologies, Ellicott City, MD, USA). Blocks of spinal cord tissue were placed directly into solutions A and B, without rinsing for 2 weeks in the dark at room temperature. Forty-eight hours after placing the blocks in solution C (4°C), they were frozen on dry ice and stored at −70°C until sectioned. Cryostat sections (100 μm) were cut at −25°C and mounted onto gelatinized slides. Slides were allowed to dry in the dark, and the rest of the staining process performed as previously described (Wallace et al., 2006). Neurons chosen for tracing met the following criteria: (1) completely impregnated with Golgi stain, (2) unobscured by other impregnated neurons or precipitant, (3) 70% of the dendritic tree was visible within the plane of focus, and (4) neurons must have been located in the outer one-half of the granule cell layer in the dentate gyrus. Cells chosen for analyses had to be well-impregnated, clearly distinguishable from adjacent cells and have continuous unbroken dendrites. Spines were counted under oil (X100), using light microscopy (Axostar Plus equipped with Axio-Cam MRc, Zeiss), and the entire visible dendritic length measured by an imaging computer program (Axio-Vision, Zeiss). Spine density was calculated referring to the length of the dendrite.

### Western blot analysis for brain-derived neurotrophic factor (BDNF), nerve growth factor (NGF), glialcellline-derived neurotrophic factor (GDNF), and neurotrophin-3 (NT-3)

Cytosolic and nuclear extracts were prepared as follows: Briefly, spinal cord tissues from each mouse were suspended in extraction Buffer A containing 0.2 mM phenylmethylsulfonyl fluoride, 0.15 μM pepstatin A, 20 μM leupeptin and 1 mM sodium orthovanadate, homogenized at the highest setting for 2 min, and centrifuged at 1000 × g for 10 min at 4°C. Supernatants represented the cytosolic fraction. The pellets containing enriched nuclei were re-suspended in Buffer B containing 1% Triton X-100, 150 mM NaCl, 10 mM TRIS-HCl pH 7.4, 1 mM EGTA, 1 mM EDTA, 0.2 mM phenylmethylsulfonyl fluoride, 20 μM leupeptin, and 0.2 mM sodium orthovanadate. After centrifugation 30 min at 15,000 × g at 4°C, the supernatants containing the nuclear protein were stored at −80°C for further analysis. The levels of BDNF, NGF, GDNF, and NT-3, were quantified in the cytosolic fraction from spinal cord tissue collected after 72 h after SCI. The filters were blocked with 1x PBS, 5% (w/v) non-fat dried milk for 40 min at room temperature and subsequently probed with specific antibodies for BDNF (1:1000), NGF (1:1000), GDNF (1:1000) in 1x PBS, 5% w/v non-fat dried milk, 0.1% Tween-20 at 4°C overnight. Membranes were incubated with peroxidase-conjugated bovine anti-mouse IgG secondary antibody or peroxidase-conjugated goat anti-rabbit IgG (1:2000, Jackson ImmunoResearch, West Grove, PA, USA) for 1 h at room temperature. To ascertain that blots were loaded with equal amounts of proteic lysates, they were also incubated in the presence of α-tubulin antibody (1:10,000, Sigma-Aldrich). Relative expression of protein bands was quantified by densitometric scanning of the X-ray films with a GS-700 Imaging Densitometer (GS-700, Bio-Rad Laboratories, Milan, Italy) and a computer program (Molecular Analyst, IBM), and standardized for densitometric analysis to α-tubulin levels.

### Statistical evaluation

All values in the figures and text are expressed as mean ± standard error of the mean (SEM) of ‘N’ observations. For *in vivo* studies, N represents the number of animals. In experiments involving immunohistochemistry, the figures shown are representative of at least three experiments performed on different days. The results of immunohistochemistry and Western blot were analyzed by one-way analysis of variance followed by a Bonferroni *post-hoc* test for multiple comparisons. BMS data were analyzed by the Mann-Whitney test. A *P*-value of less than 0.05 was considered significant (Paterniti et al., 2013a).

## Results

### Co-ultraPEALut treatment promotes functional recovery

**Figure 1:**
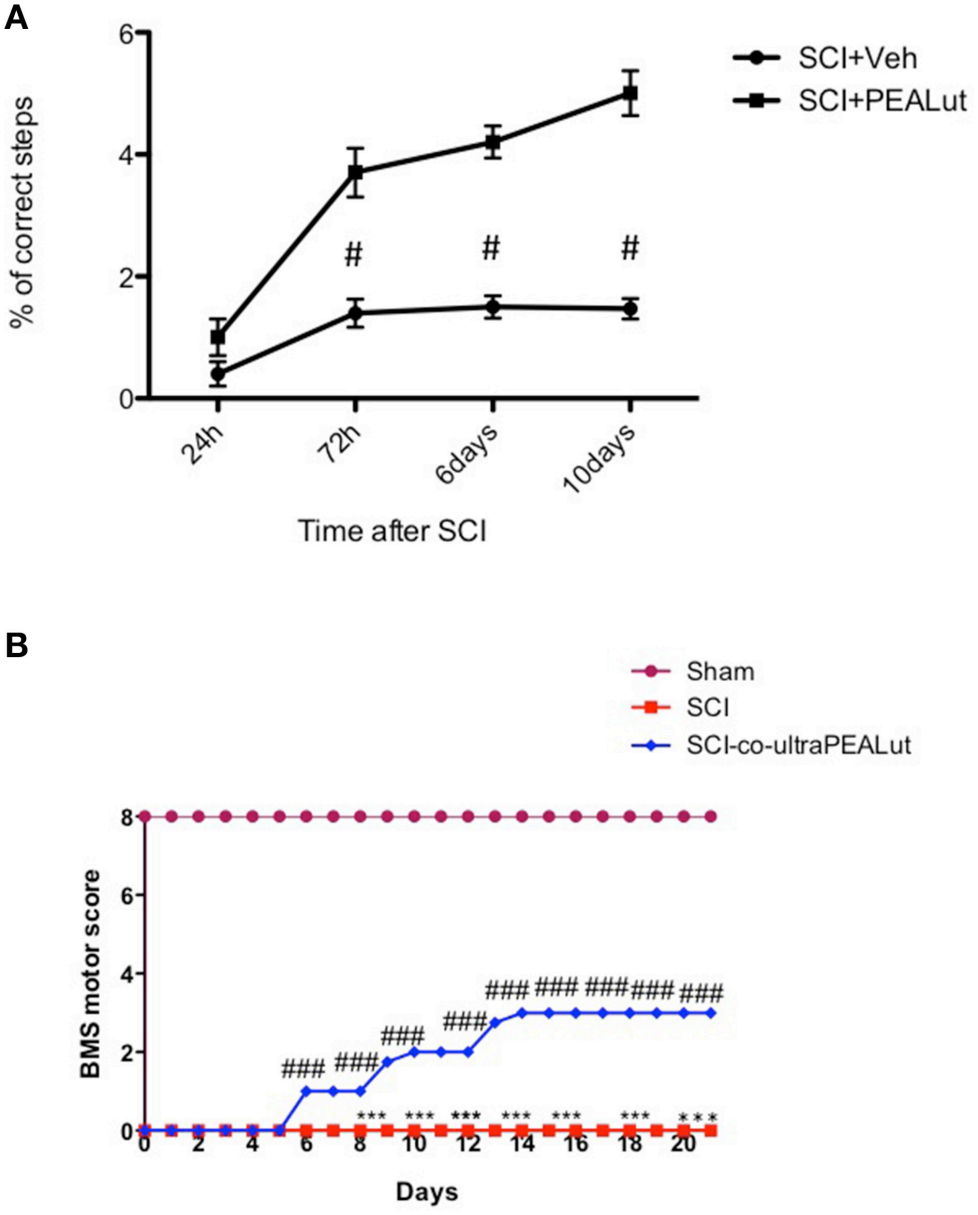
**Evaluation of co-ultraPEALut on motor function after SCI. (A)** Grid walking improved significantly 72 h after co-ultraPEALut administration; **(B)** The functional recovery was assessed every day until 21 days after SCI by Basso Mouse Scale (BMS) open-field score. Administration of co-ultraPEALut reduced the motor disturbance after SCI. Values are mean ± SEM (*N* = 10 per group). (^#^*p* < 0.05 vs. SCI group; ^*###*^*p* < 0.001 vs. SCI group).

Co-ultraPEALut treatment, in an earlier study, improved motor activity in SCI compared to other treatments 10 days after injury (Paterniti et al., 2013b). In confirmation of this result, treatment with co-ultraPEALut significantly ameliorated motor performance10 days post-injury, as evidenced in a horizontal grid walking test in which fewer missteps of the left hind limb during grid walking were detected (Figure 1A). Furthermore, in order to evaluate loss of motor function associated with spinal cord damage, a BMS score was employed to assess daily the functional recovery until 21 days after injury. SCI mice showed a significant deficit in movement which improved following co-ultraPEALut administration (Figure 1B).

### Effect of co-ultraPEALut administration on cell proliferation

**Figure 2:**
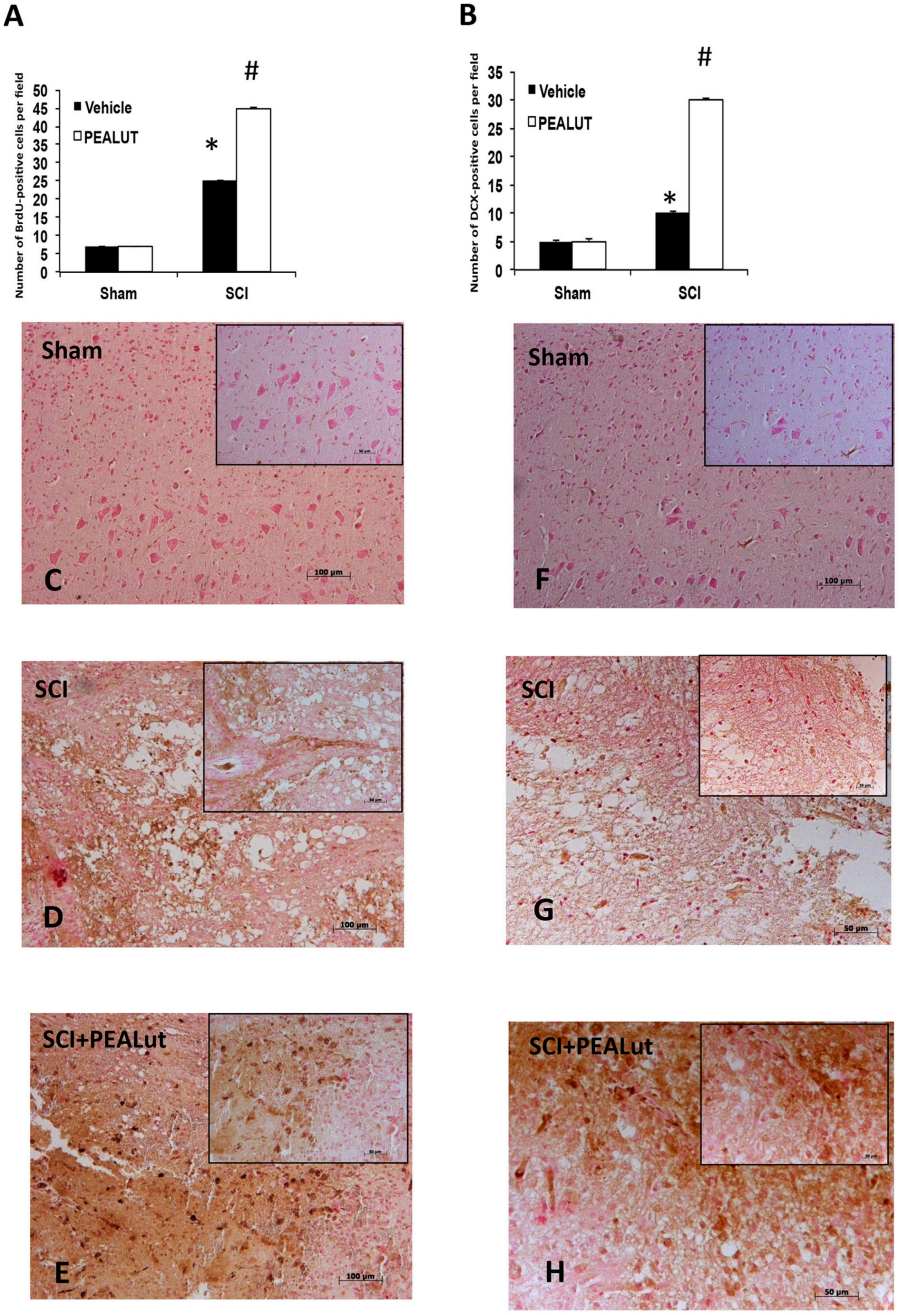
**Effect of co-ultraPEALut on cell proliferation in spinal cord of SCI mice. (A)** BrdU (70 mg/kg, i.p.) was given 2 h before sacrifice to examine the effects of 72 h co-ultraPEALut administration. In SCI-operated mice the number of proliferating cells increased at 72 h. Moreover, co-ultraPEALut treatment further increased this number. Values are mean ± SEM (*N* = 10 per group). (^*^p < 0.05 vs. sham group; ^#^*p* < 0.05 vs. SCI group). **(C)** BrdU^+^ nuclei in sham group **(D)**, BrdU^+^ nuclei in SCI-operated mice **(E)**, BrdU^+^ nuclei in co-ultraPEALut treatment. **(B)** In SCI-operated mice the number of DCX^+^ cells increased at 72 h; the number of DCX^+^ cells is increased after co-ultraPEALut treatment. Values are mean ± SEM (*N* = 10 per group; ^*^p < 0.05 vs. sham group; ^#^*p* < 0.05 vs. SCI group). **(F)** DCX^+^ cells in sham group at 20X magnification, **(G)** DCX^+^ cells in SCI-operated mice **(H)** DCX^+^ cells in co-ultraPEALut treatment group. All images are at 20X magnification.

**Figure 3:**
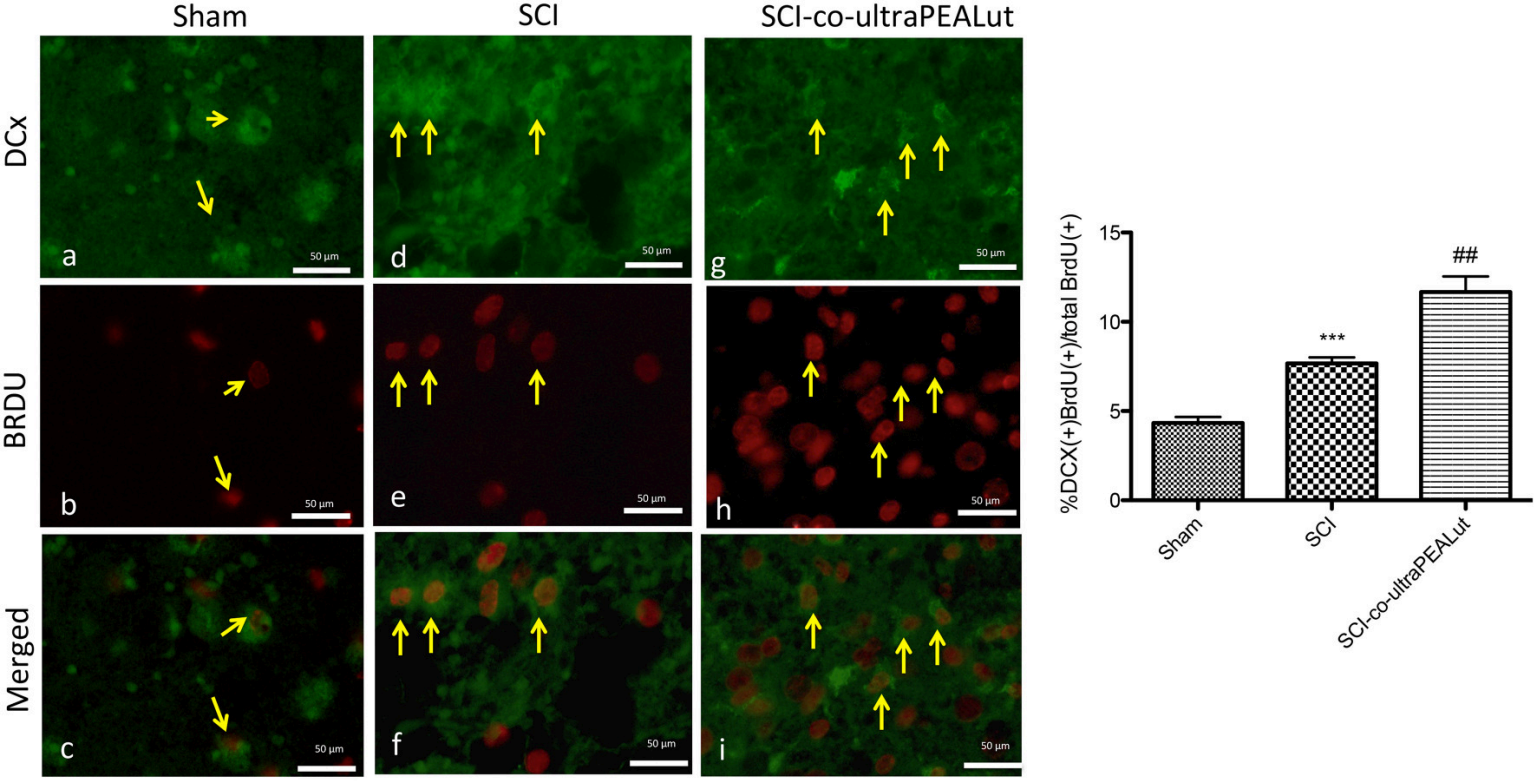
**Effect of co-ultraPEALut on co-localization of BrdU/DCX labeling after SCI**. Results are shown for **(A–C)** sham-operated mice, **(D–F)** mice with SCI, and **(G–I)** mice with SCI treated with co-ultraPEALut. Spinal cord lesions sections, obtained from perilesioned area, were double-stained with antibodies against BrdU and DCX. The staining revealed that the increased proliferation of new neurons (DCX^+^ cells) started in mice subject to SCI **(D–F**, see yellow arrows that indicate the overlay) but treatment with co-ultraPEALut significantly increased the proliferartion after 72 h **(G–I**, see yellow arrows), see densitometric analysis. All images were digitalized at a resolution of 8 bits into an array of 2048 × 2048 pixels. (^*##*^*p* < 0.01 vs. SCI; ^***^p < 0.001 vs. sham).

Diffuse cellular proliferation throughout the gray and white matter has been observed in different models of SCI (Kozlova, 2003; Zai and Wrathall, 2005). In order to identify the cell type(s) undergoing proliferation and to evaluate whether they migrate as a result of injury, animals were injected with BrdU to label dividing cells in the S phase of the cell cycle. Cellular alterations in the peri-lesioned zone (boundary between the core necrotic area and penumbra) and in regions distal to the lesion (identified by counting five optical fields on both sides from the core lesion) were assessed by counting the cell populations labeled with antibodies against BrdU, DCX, NeuN, GFAP, and MAP2. Proliferation and numbers of immature neurons were determined using BrdU and DCX immunolabelling, respectively (Malberg et al., 2000). Figures 2A,B revealed a notable difference in the number of BrdU^+^ nuclei and DCX^+^ cells between sham, SCI-operated mice and SCI mice treated with co-ultraPEALut. Numbers of both BrdU^+^ nuclei and DCX^+^ cells increased 72 h after SCI to a greater extent with co-ultraPEALut administration (Figures 2C–H). Moreover, there was an increased expression of immunofluorescence staining of DCX^+^ cells after treatment with co-ultraPEALut in SCI mice (Figure 3) and comparable to control levels of sham mice (Figure 3).

**Figure 4:**
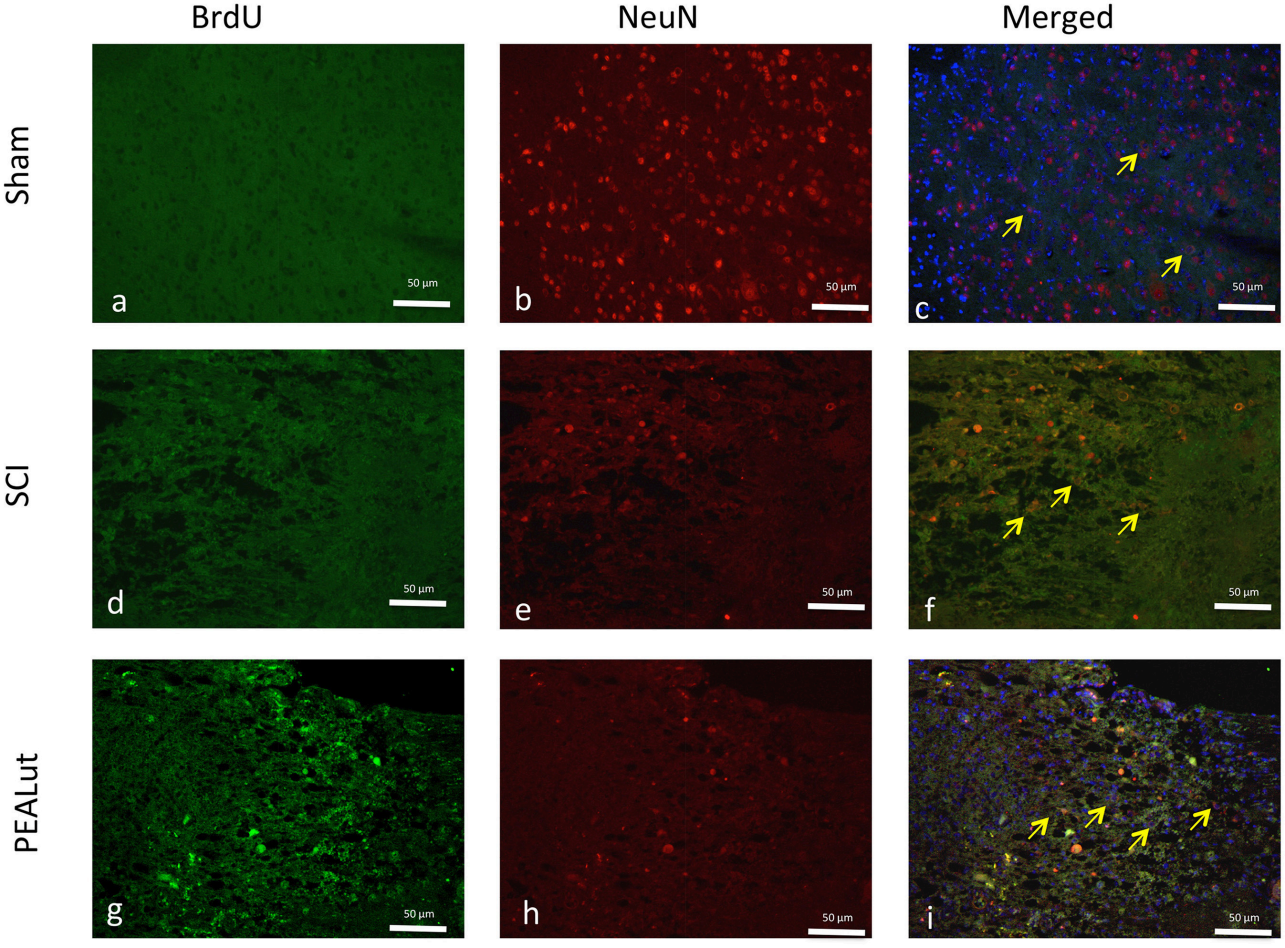
**Effect of co-ultraPEALut on co-localization of BrdU/NeuN after SCI**. Results are shown for **(A–C)** sham-operated mice, **(D–F)** mice with SCI, and **(G–I)** mice with SCI treated with co-ultraPEALut. Spinal cord lesion sections, obtained from perilesioned area, were double-stained with antibodies against BrdU and NeuN. NeuN expressin increased in the group treated with co-ultraPEALut (**G–I**, see yellow arrows). All images were digitalized at a resolution of 8 bits into an array of 2048 × 2048 pixels.

**Figure 5:**
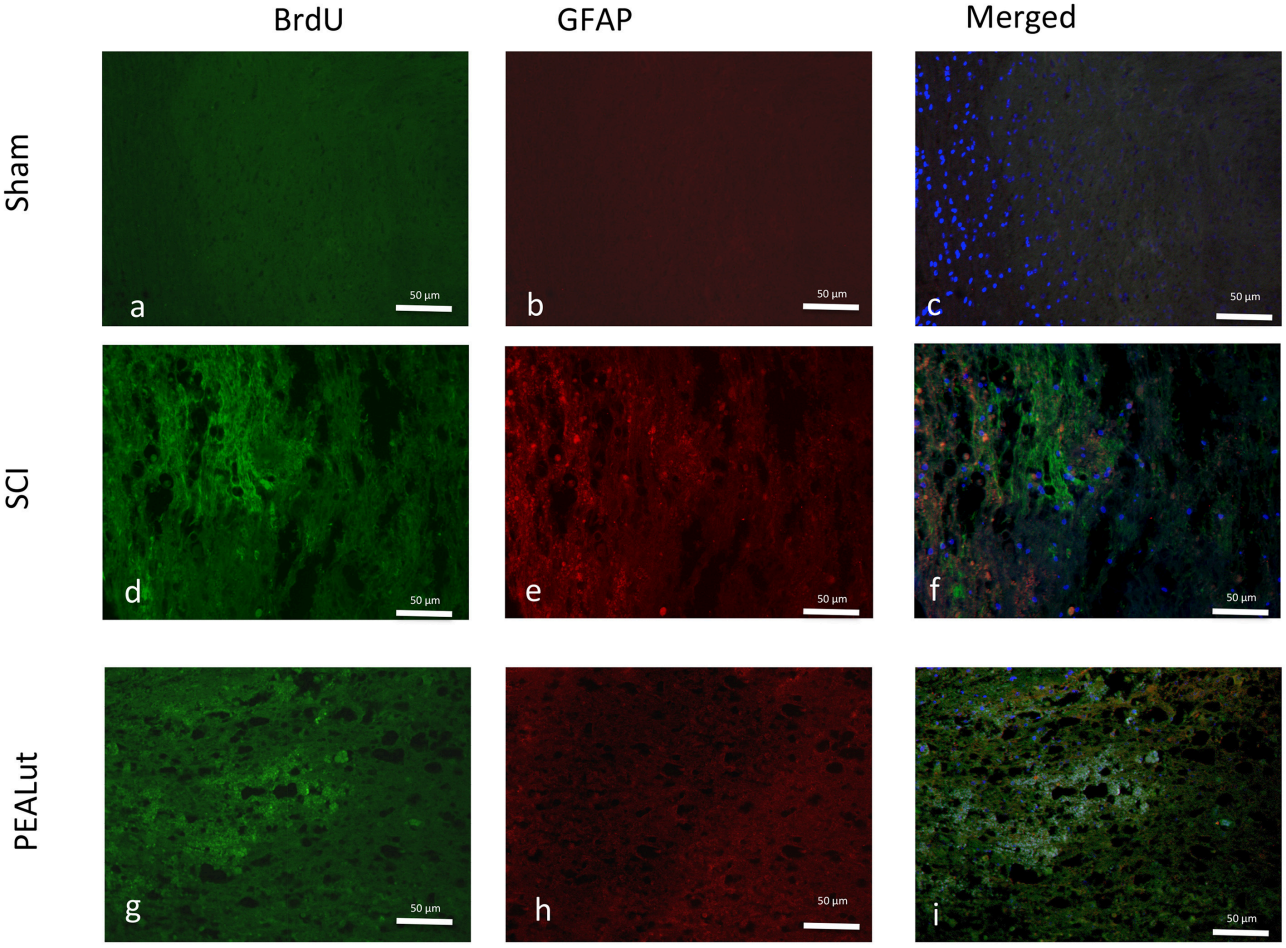
**Effect of co-ultraPEALut on co-localization of BrdU/GFAP after SCI**. Results are shown for **(A–C)** sham-operated mice, **(D–F)** mice with SCI, and **(G–I)** mice with SCI treated with co-ultraPEALut. Spinal cord lesion sections, obtained from perilesioned area, were double-stained with antibodies against BrdU and GFAP. Spinal cord sections revealed increased astrogliosis (GFAP^+^ cells) in SCI mice **(D–F)**. All images were digitalized at a resolution of 8 bits into an array of 2048 × 2048 pixels.

To explore further the nature of these proliferating cells, we co-stained sections with the anti-BrdU antibody and antibodies against either NeuN or GFAP, and calculated the number of double-positive cells near the lesion borders. Seventy-two hours after SCI, a significant percentage of NeuN^+^ and GFAP^+^ cells showed BrdU incorporation Figures 4A,B. Representative images showing the increase of proliferating NeuN^+^ and GFAP^+^ cells compared to sham animals are shown in Figures 4, 5.

**Figure 6:**
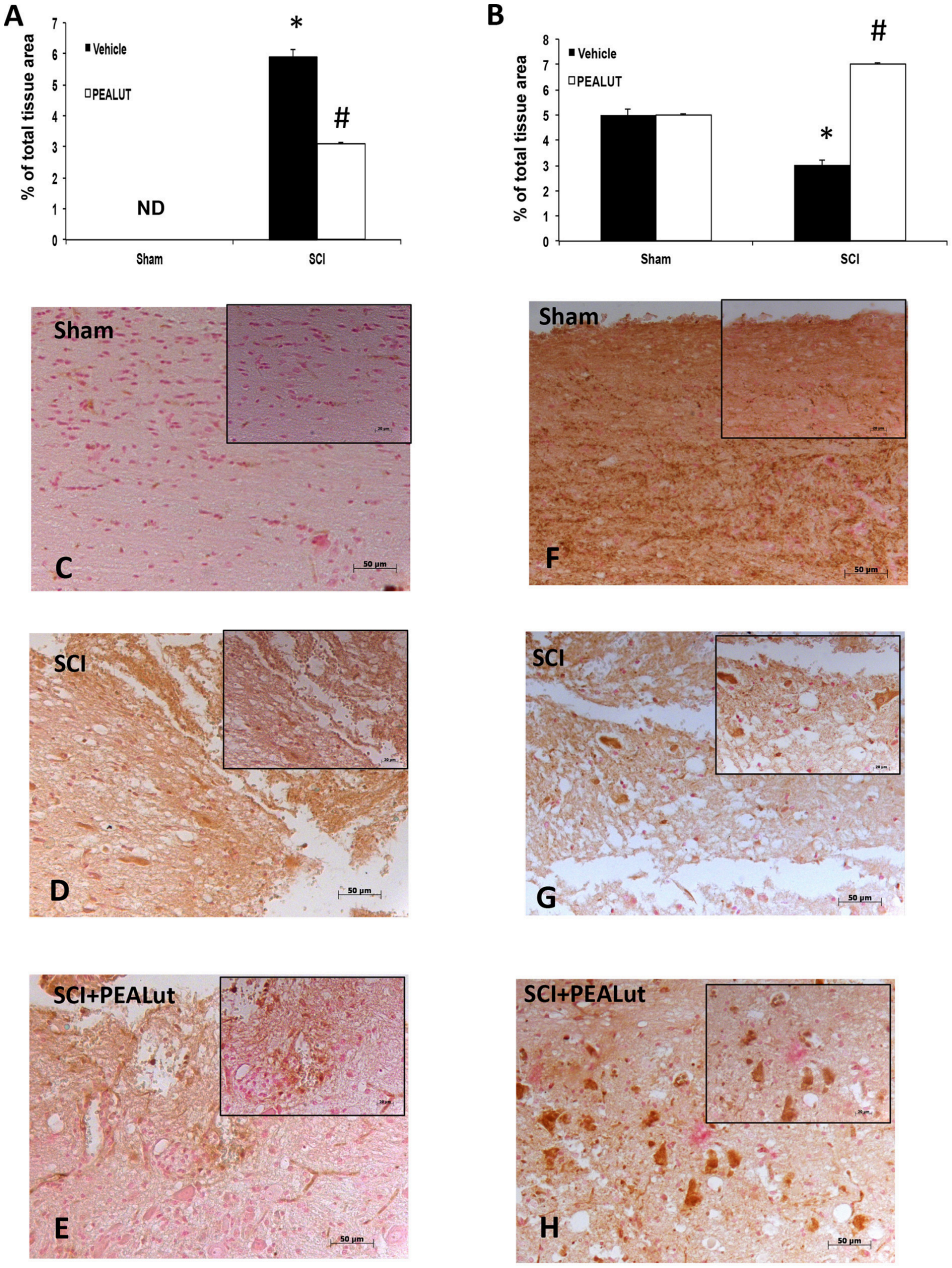
**Effect of co-ultraPEALut on GFAP and MAP-2 expression in spinal cord of mice subjected to SCI. (A)** In SCI-operated mice the number of GFAP^+^ cells increased at 72 h; the number of GFAP^+^ cells decreased after co-ultraPEALut treatment. Values are mean ± SEM (*N* = 10 per group; ^*^p < 0.05 vs. sham group; #*P* < 0.05 vs. SCI group). **(C)** GFAP^+^ cells in sham group, **(D)** GFAP^+^ cells in SCI-operated mice, **(E)** GFAP^+^ cells in co-ultraPEALut treated mice. **(B)** In SCI-operated mice the number of MAP2^+^ cells decreased at 72 h; co-ultraPEALut treatment increased the number of MAP2^+^-expressing cells. Values are mean ± SEM (*N* = 10 per group; ^*^p < 0.05 vs. sham group; ^#^*p* < 0.05 vs. SCI group). **(F)** MAP2^+^ cells in sham group, **(G)** MAP2^+^ cells in SCI-operated mice, **(H)** MAP2^+^ cells in co-ultraPEALut treated mice. All images are at 20X magnification. The image were obtained from perilesioned area.

Qualitative analysis of spinal cord sections showed pronounced astrogliosis (GFAP^+^ cells) in the peri-lesional area after SCI (Figure 6A), which was significantly diminished in the spinal cord from co-ultraPEALut-treated SCI mice (Figure 6A). In terms of MAP2-expressing (neuronal) cells, there was a significant reduction after 72 h; in contrast, treatment with co-ultraPEALut at 72 h largely prevented this decrease (Figure 6B). Representative images showing the increase in proliferating GFAP^+^ and decrease in MAP2^+^ cells compared to sham animals is shown in Figures 6C–H.

### Effect of co-ultraPEALut on neurotrophic factor levels

**Figure 7:**
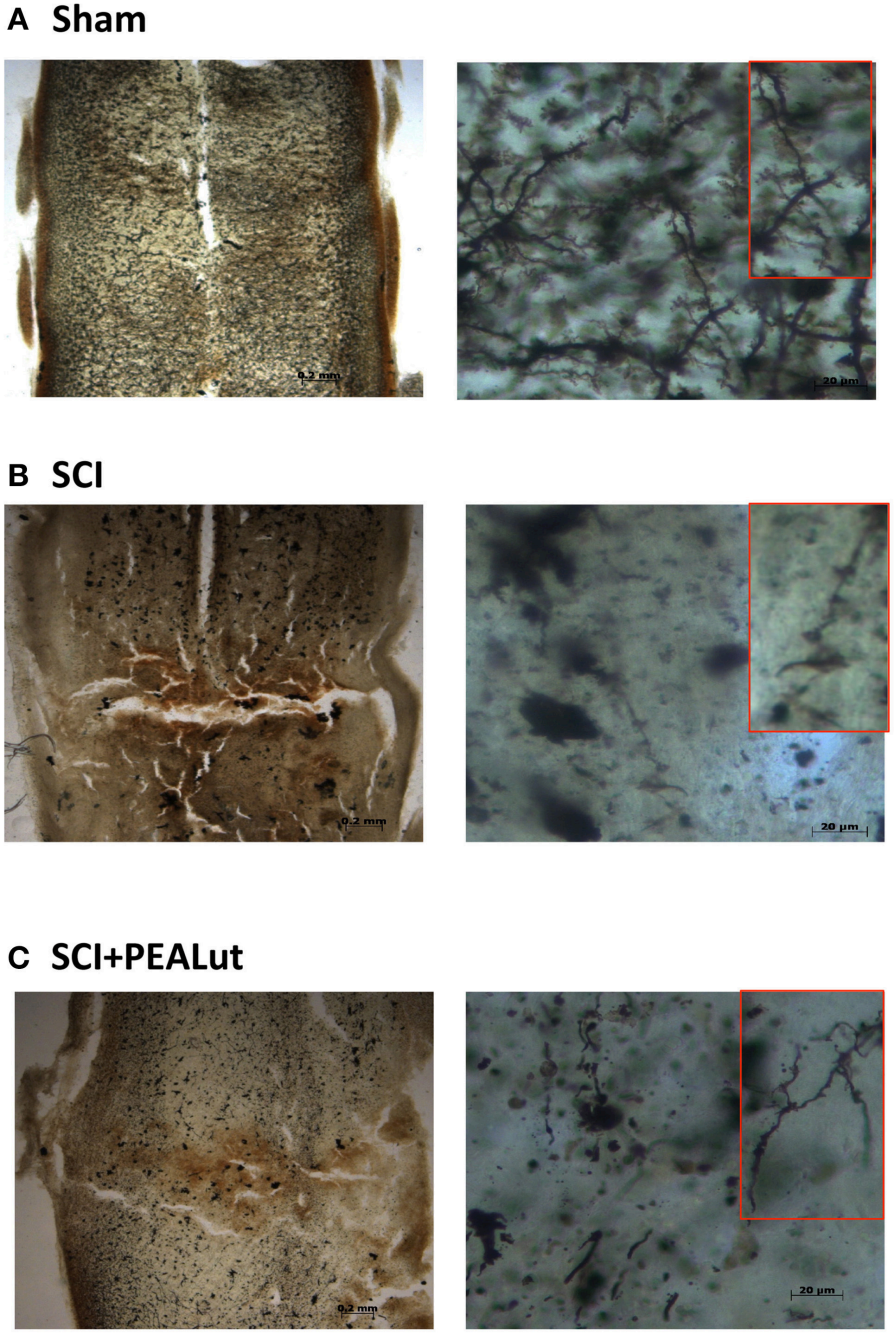
**Effect of co-ultraPEALut on dendritic remodeling and spine density in spinal cord of mice subjected to SCI. (A–C)** Effects of co-PEALut administration on spine density; qualitative analysis of spine density showed an increase in mice treated with co-ultraPEALut.

**Figure 8:**
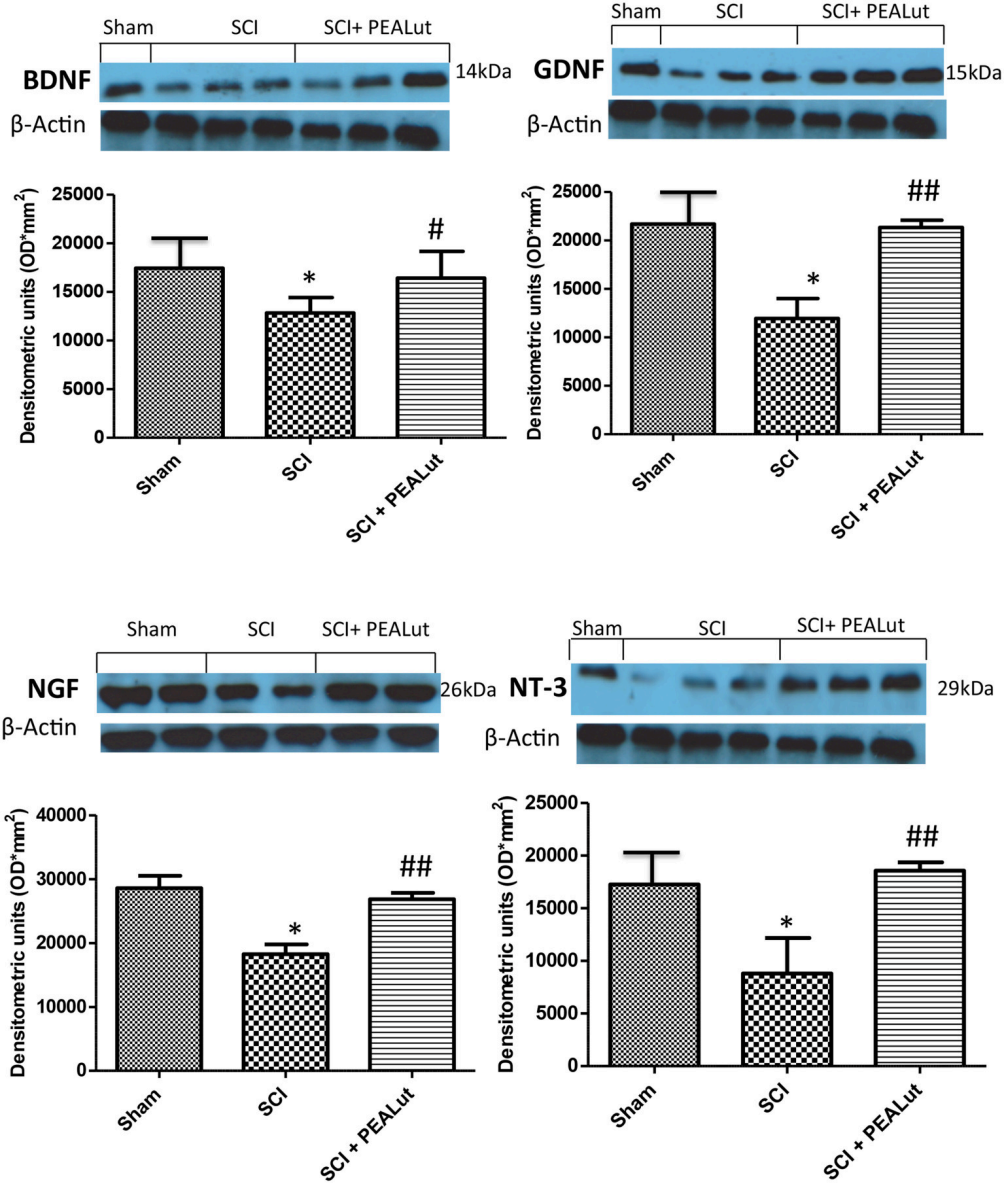
**Effect of co-ultraPEALut on neurotrophin levels in spinal cord of mice subjected to SCI**. Significant increases in BDNF, GDNF, NGF, NT-3 levels were observed in spinal cord of SCI mice treated with co-ultraPEALut treatment. β-actin was used as internal control. A representative blot of lysates obtained from each group is shown, and densitometric analysis of all animals is reported (*N* = 10 mice from each group; ^*^p < 0.05 vs. sham group; ^#^*p* < 0.05 vs. SCI group; ^*##*^*p* < 0.01 vs. SCI group).

To correlate the neurogenic effect of co-ultraPEALut treatment on dendritic remodeling and reorganization, a Golgi staining method was employed. Our findings showed a significant disruption in dendritic organization with a loss of synaptic bottoms in tissue collected 72 h after SCI. Co-ultraPEALut administration stimulated the remodeling of dendritic arbors in the injured area (Figure 7). To assay whether co-ultraPEALut modulates the neuroregenerative process through regulation of neutrophic factors, we analyzed BDNF, GDNF, NGF, and NT-3 protein levels in the peri-lesioned zone by Western blot. Spinal cord tissues harvested 72 h after trauma exhibited significantly diminished neurotrophic factor expression compared to sham animals (Figure 8). Co-ultraPEALut administration (Figure 8, and densitometric analysis) remarkably re-established the expression of these neurotrophic factors, reaching levels seen in uninjured mice.

## Discussion

The discovery of neurogenesis in the adult mammalian CNS represents a potential paradigm shift in regenerative medicine and a therapeutic weapon for CNS injuries. Numerous studies have addressed this regenerative process close to the lesion site in an attempt to understand if newly generated neurons could replace those dying within the damaged tissue. Regions considered distal to the lesion have received little attention until now, although it is conceivable that a focal injury could alter unaffected CNS areas. Investigating the neuroregenerative properties of co-ultraPEALut in a murine model of SCI, we now show that co-ultraPEALut increased both BrdU^+^ nuclei and DCX^+^ cells in the spinal cord of injured mice, stimulated expression of the neurotrophic factors BDNF, GDNF NGF, and NT-3, promoted dendritic remodeling and preserved spine density.

Focal CNS damage leads to acute loss of function and neurodegeneration, which is followed by a regenerative response in an attempt to re-establish structure and function. Primary injury to the spinal cord is classified as an irreversible event that begins with an unexpected, traumatic blow to the spine provoking local segmental cord damage, which is followed by a second phase of tissue degeneration that can occur over weeks or months. The latter is characterized by glutamate excitotoxicity, release of pro-inflammatory cytokines, and oxidative stress. These processes take part in a cascade culminating in dysfunction and death of neurons (Anderson and Hall, 1993). This inflammatory process is followed by production of free radicals and nitric oxide (Genovese et al., 2006b). Therapies targeting those factors implicated in the secondary degeneration cascade lead to tissue sparing and improved neurological outcomes in SCI-lesioned animals (Cuzzocrea et al., 2006; Genovese et al., 2006a). Spinal immobilization is still considered the standard prehospital care for SCI patients. Many pharmacological treatments have been tested in SCI, but none have met substantial success. High-dose corticosteroids, given within the first 8 h after injury and over the next 24–48 h remains part of the current treatment regimen (Bracken et al., 1998), although recent findings have come to question the effectiveness of corticosteroids in SCI. Perhaps not surprisingly, the limited success of these clinical studies reflect the complexity of the secondary degenerative response in SCI (Leker and Shohami, 2002). Because such therapies address only one aspect of this response, a successful treatment might require a multimodal approach (King et al., 2006).

PEA possesses both anti-inflammatory and neuroprotective effects (Paterniti et al., 2013a), but lacks antioxidant activity. We thus decided to test the activity of a co-ultramicronized composite containing PEA and luteolin (“co-ultraPEALut”). We previously described the neuroprotective and neuroinflammatory properties of co-ultraPEALut in a mouse SCI model (Paterniti et al., 2013b), as well as the capacity of this formulation to promote neurogenesis and neuroplasticity in a mouse model of a depression-like state (Crupi et al., 2013). Precursor cells in the main neurogenic areas of adult brain and local progenitors play a prominent role in recovery after CNS injury (Butti et al., 2012). The hippocampal SVZ and SGZ are responsible for most neurogenic activity occurring in adult mammals (Doetsch, 2003). In these areas, ischemic damage stimulates neurogenesis, where a pool of progenitors is produced up to 4 months post-injury (Thored et al., 2006). In the regenerating tissue, new neurons and neuroblasts proliferate, and migrate in chains along blood vessels toward the ischemic area to provide trophic support (Thored et al., 2007). Neurogenesis and neovascularization in the injured CNS are considered interdependent processes that share common mediators and signals (Snapyan et al., 2009). At 72 h post-injury co-ultraPEALut treatment stimulated the proliferation of mature neurons, as demonstrated by the number of DCX^+^ cells. Further, quantitative analysis of spine density in spinal cord revealed that both spine and dendritic morphology were sensitive to co-ultraPEALut treatment. MAP2 participates in neuronal development, structural stability, apophysis formation, and regulation of synaptic plasticity. In the CNS, MAP2 interacts with microtubules to promote their assembly and stability and is associated with axonal transport (Okabe and Hirokawa, 1989). Because MAP2 is exclusively only in dendrites and neurites within the CNS, it is considered a molecular marker of neurons (Rioux et al., 2003; Furutani and Kibayashi, 2012). Interestingly, MAP2 expression increased in response to co-ultraPEALut administration, strengthening the view that this agent is capable of stimulating regeneration and remodeling spinal structure after damage.

Neurotrophins such as BDNF, NGF, or NT-3 are well-known to promote regeneration of injured nerves (Nakagawara et al., 1994; Pezet and Malcangio, 2004; Song et al., 2008). The present study showed that levels of BDNF, GDNF, NGF, and NT-3 were down-regulated by spinal cord damage, while a sustained increase in their expression in peri-lesioned tissue was brought about following administration of co-ultraPEALut. Treatments which regulate endogenous synthesis of neurotrophins may be superior to their exogenous administration, as these proteins are unable to cross the blood–brain barrier and, in the case of BDNF may encounter difficulties in CNS diffusion caused by the truncated TrkB receptor in astroglia (Biffo et al., 1995; Song et al., 2008). It is tempting to speculate that the observed increase in neurotrophic factor expression may account, at least in part, for the neuroprotective activity of co-ultraPEALut. Moreover, it is conceivable that the neurogenic effect of co-ultraPEALut is linked to neurotrophic factor expression.

The endocannabinoid system regulates a broad range of processes such as axonal growth and guidance during development (Berghuis et al., 2007), adult neurogenesis (Goncalves et al., 2008), and numerous behavioral responses associated with endocannabinoid retrograde signaling at synapses. The endocannabinoid system is regulated by neurological insults such as cerebral ischemia, traumatic and focal brain injury, and it is increasingly considered a promising therapeutic target in many CNS pathologies (Velayudhan et al., 2014) including SCI (Adhikary et al., 2011). Endocannabinoid release differs from that of canonical neurotransmitters which are synthesized and stored in synaptic vesicles; rather, they are generated from membrane precursors and released into the synaptic cleft following neuronal activation (“on demand”). Different mechanisms have been proposed to for the mechanism of PEA action (Loverme et al., 2005), including interaction with uncharacterised cannabinoid receptor 2-like receptors, the peroxisome proliferator-activated receptor-α, and inhibition of FAAH (fatty acid amide hydrolase), thus increasing local concentrations of anandamide and probably PEA (the so-called “entourage effect”; Lambert et al., 2002). The actions of anandamide, 2-arachidonylglycerol and PEA may be prolonged by FAAH inhibition. Inhibition of FAAH strengthens the pharmacological effects of anandamide and PEA *in vivo*. Flavonoids possess many pharmacological properties, including anti-inflammatory and cytoprotective. For example, apigenin, which is stucturally related to luteolin, protects against endoplasmic stress-induced neuronal cell death (Choi et al., 2010). Here, we utilized the endocannbinoid congener PEA and the flavonid luteolin as an ultramicronized composite to protect from neurodegeneration in a mouse model of SCI. We have previously demonstrated that PEA administration (10 mg/kg) exerts anti-inflammatory and neuroprotective effects in a mouse model of SCI (Esposito et al., 2011). Whether or not luteolin cooperates with PEA in one or more of these mechanisms is a fascinating question to be explored in future studies.

## Conclusions

Translation of experimental results into clinical practice has been particularly challenging when dealing with neuroregeneration. Nevertheless, our studies show that co-ultraPEALut exerts a spectrum of actions: neuroprotection, neuroregeneration, anti-inflammation, and anti-apoptosis. The present findings reinforce recent data demonstrating that co-ultraPEALut can ameliorate symptomatology of diseases such as SCI in animals. Therefore, we propose that co-ultraPEALut administration can be considered as a novel therapeutic approach in the treatment of neurological outcome after SCI. This composite was administered after trauma, in an environment that simulates the clinical condition, and therefore may have clinical implications.

## Author contributions

RC drafted the manuscript, participated in research design, and carried out immunohistochemical analysis; DI participated in research design and carried out the *in vivo* experiments; GB carried out the *in vivo* experiments; MC and IP performed immunohistochemical analysis; RS performed Western blot analysis; EE analyzed the data; SC and EE contributed to writing the manuscript and designing the experiments. All authors read and approved the final manuscript.

## Funding

This study was supported by the Italian Ministry of Instruction, University and Research grants (MIUR; PON01_02512 grant).

### Conflict of interest statement

SC, researcher on the study team, is co-inventor on patent WO2013121449 A8 (Epitech Group SpA) which deals with compositions and methods for the modulation of amidases capable of hydrolysing N-acylethanolamines useable in the therapy of inflammatory diseases. Moreover, SC is also a co-inventor with Epitech group on the following patent: (1) EP 2 821 083, (2) MI2014 A001495, (3) 102015000067344. None of these patents are relative to the ongoing study. The remaining authors declare that the research was conducted in the absence of any commercial or financial relationships that could be construed as a potential conflict of interest.
